# Sarcoma With Pulmonary Metastases: A Management Dilemma

**DOI:** 10.7759/cureus.65344

**Published:** 2024-07-25

**Authors:** Juin Yi Ng, Nurul Amirah Ahamed Siddeekh, Muhammad Ishamuddin Ismail, Mohd Ramzisham B Abdul Rahman, Nur Ayub Md Ali

**Affiliations:** 1 Cardiothoracic Surgery, Hospital Canselor Tuanku Muhriz, Kuala Lumpur, MYS

**Keywords:** palliative, pulmonary metastasectomy, aggressive cancer, sarcoma with lung metastasis, osteosarcoma, soft tissue sarcoma

## Abstract

Pulmonary metastases in soft tissue, such as sarcoma and osteosarcoma, are associated with a poor prognosis. A complete surgical resection has been proven to prolong survival. We report four cases of sarcoma with pulmonary metastases, all with differing progressions, prognoses, and management. This highlights the challenging nature of managing sarcoma with pulmonary metastases. Surgical metastatectomy remains the mainstay treatment for sarcoma with pulmonary metastases. Studies have demonstrated a significant survival benefit with complete surgical resection. There is currently no consensus on the size of the metastasis or the number of lesions for considering a patient inoperable. Surgical metastatectomy provides improved survival for sarcoma patients with pulmonary metastases. Management strategy is rapidly evolving with the emergence of new treatment methods. A case-by-case assessment and MDT approach are paramount in deciding the best course of action.

## Introduction

Sarcomas are a heterogenous group of mesenchymal neoplasms that can be generally categorized into soft tissue sarcoma and osteosarcoma. Pulmonary metastases commonly occur with osteosarcoma, but they may occur with any sarcoma [1]. Sarcomas have the potential to metastasize or spread to other parts of the body, with the lungs being one of the most common sites of metastasis in sarcoma. In osteosarcoma with pulmonary metastases, its incidence follows a bimodal age distribution, with two dominant peaks in adolescent and elderly patients [2]. Pulmonary metastases are a significant concern in patients with sarcoma and are associated with a particularly poor prognosis. It is estimated that approximately 20% of sarcoma patients have detectable metastasis at the time of diagnosis [3]. The five-year overall survival for osteosarcoma patients with lung metastases is approximately 30%, in contrast to 70% for those without metastasis [4].

Patients with pulmonary metastases from sarcoma are currently treated with complete surgical resection, and the same chemotherapy regimen is recommended for patients with high-grade sarcoma that is localised (three cycles of cisplatin and doxorubicin as neoadjuvant chemotherapy, followed by an additional eight alternating cycles of ifosfamide, etoposide, and cisplatin doxorubicin) [5]. Even with combined therapy, the prognosis for metastatic disease is still dismal, with over 50% of cases relapsing. It is still unclear what the optimal course of action is for treating patients with lung metastases from sarcoma. Enhancing comprehension of the risk and clinicopathological characteristics of patients with pulmonary metastases from sarcoma can aid in identifying high-risk patients and lead to a better prognosis. This highlights the critical need for effective management strategies tailored to address the challenges posed by pulmonary metastases in sarcoma. In this article, we share our experience managing a series of patients who presented with pulmonary metastases secondary to sarcoma.

## Case presentation

Case 1

An 18-year-old male patient arrived with a swollen left leg following a minor injury. The swelling was gradually enlarging, and magnetic resonance imaging (MRI) of the left lower limb showed a large posterior leg intramuscular mass with local infiltration and a mass effect. A biopsy of the lesion showed left tibia telangiectatic osteosarcoma with peroneal nerve involvement. Contrast-enhanced computed tomography (CECT) of the thorax, abdomen, and pelvis showed multiple tiny lung nodules measuring less than 3 mm. Despite undergoing neoadjuvant chemotherapy of doxorubicin and cisplatin for three cycles, repeated MRI showed worsening enlarging posterior leg intramuscular mass with mass effect. The patient then underwent left above-knee amputation (AKA) and chemotherapy with doxorubicin and cisplatin for three cycles.

**Figure 1:**
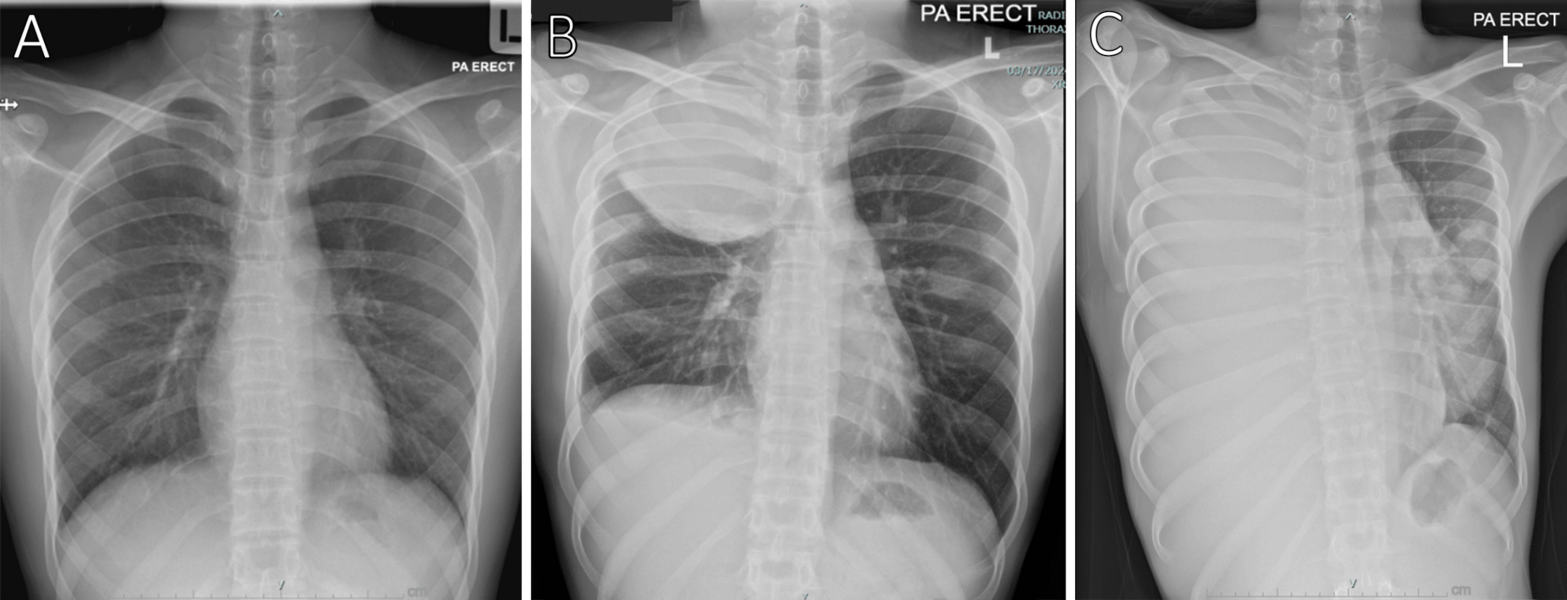
Serial chest radiography showing the rapid progression of the lung tumour upon first encounter (A), three weeks later (B), and seven weeks later (C).

**Figure 2:**
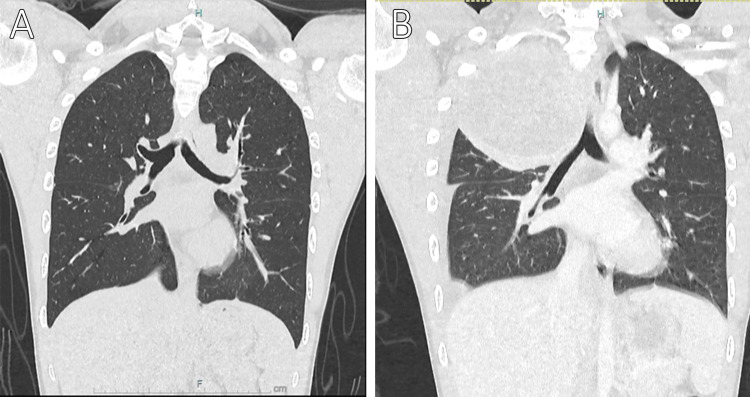
Serial contrast-enhanced computed tomography (CECT) from first encounter (A) and three weeks later (B) showing now a large heterogeneously enhancing mass occupying the right upper lobe, compressing onto the right upper lobe bronchus.

A follow-up CECT scan revealed the presence of new pleural metastases in addition to the previously identified lung nodules. The patient was counselled for pulmonary metastasectomy (PM). Unfortunately, repeated chest radiography upon admission for the procedure three weeks later revealed a rapidly enlarging lung tumor (Figure 1). A repeated chest CECT revealed numerous new, larger lung and pleural-based nodules in accordance with increasing lung and pleural metastasis adhering tightly to the main arteries and major airways; the lesion size of the right upper lobe expanded significantly to 10 cm × 9 cm × 10 cm (Figure 2). Palliative care was recommended after a multi-disciplinary team discussion because the lesions were not feasible for chemotherapy or surgery. The patient succumbed to his death one month later, sadly. Posthumously, the histopathological analysis confirmed the diagnosis of metastatic osteosarcoma.

Case 2

**Figure 3:**
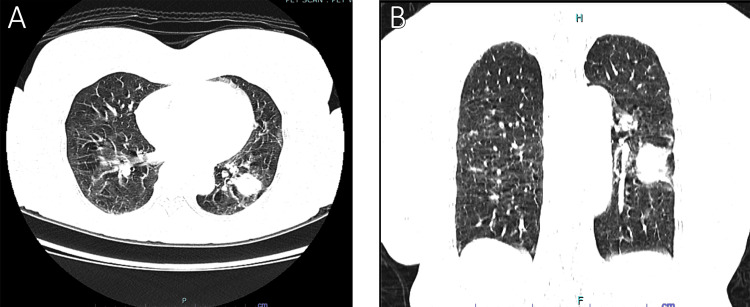
Axial view (A) and coronal view (B) of the CT thorax showed a single lung nodule over the left lower lobe.

A 61-year-old lady was diagnosed with recurrent thigh epithelioid sarcoma that was not responsive to adjuvant chemotherapy and has recurred twice. She underwent multiple wide local excisions of the tumor and reconstruction of her right thigh. Intraoperatively, the mass was resected with a clear margin and was confirmed histologically as epithelioid sarcoma. A computed tomography (CT) scan and positron emission tomography (PET) scan showed an enlarging single lung nodule over a span of four years, measuring approximately 2.0 cm × 2.9 cm × 2.1 cm (AP × W × CC) (Figure 3). A CT-guided lung biopsy of the left lower lobe showed metastatic high-grade sarcoma. She successfully underwent left video-assisted thoracoscopic surgery (VATS) and metastasectomy of the left lung lesion. She recovered well post-operatively and was discharged well.

Case 3

**Figure 4:**
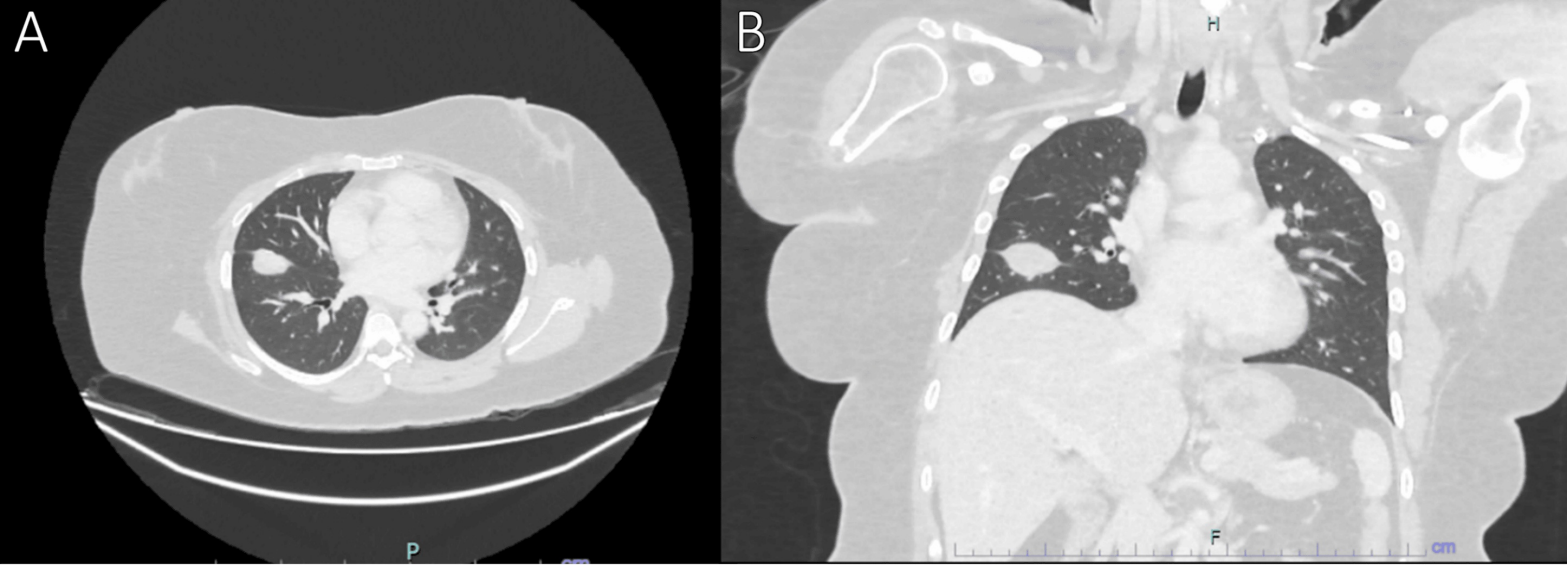
Axial view (A) and the coronal view (B) of the CT scan showed the largest lung mass seen at the right oblique fissure.

**Figure 5:**
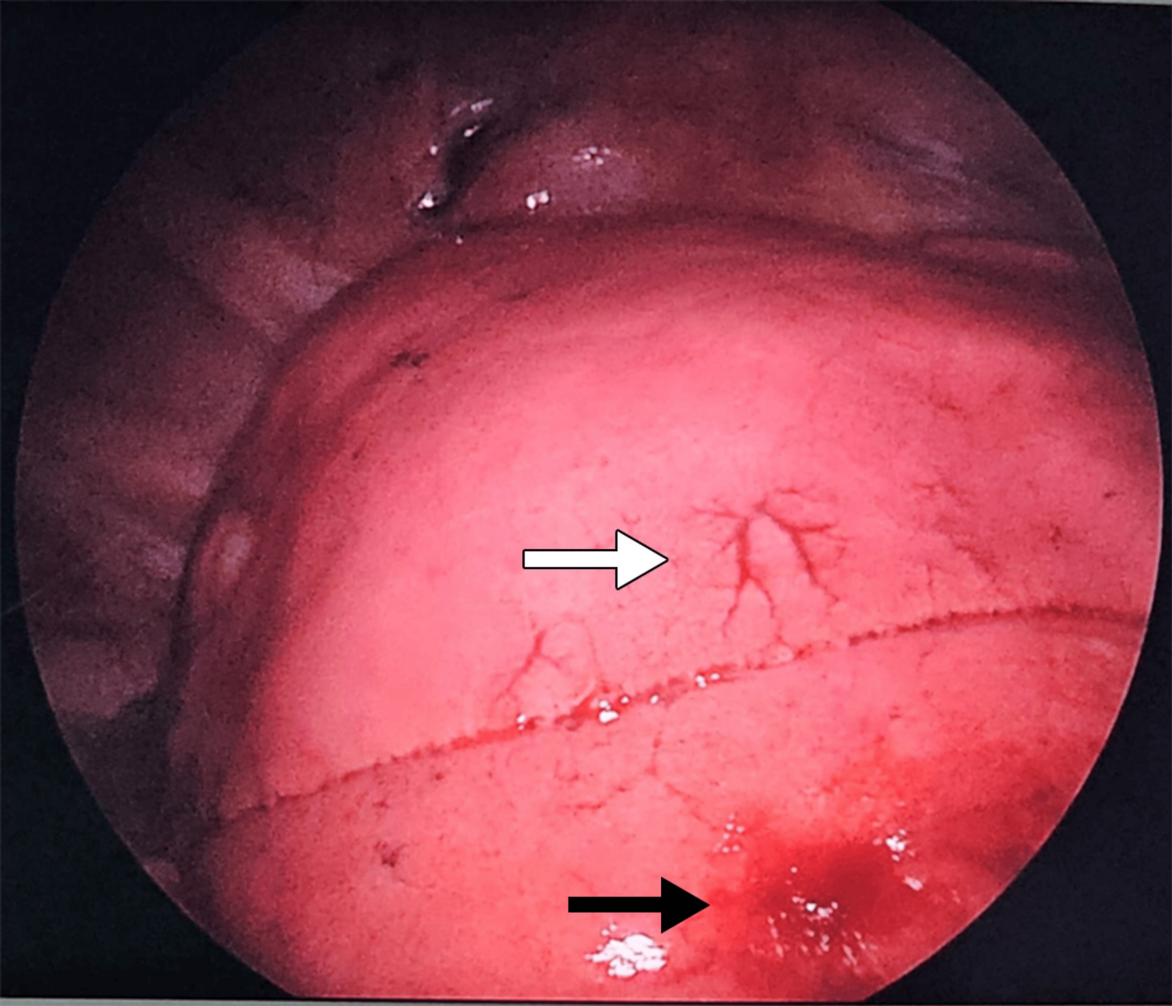
Intraoperative findings of new mass on the lateral surface of the left lower lobe (black arrow) and one of the telangiectatic vessels seen (white arrow).

A 40-year-old lady has a previous history of right elbow synovial sarcoma, which was operated on and radiotherapy completed 25 years ago. She defaulted on her follow-up thereafter and presented back when it recurred aggressively at the distal humerus with intraarticular extension, as shown by the MRI. A PET scan showed no distant metastasis. She underwent right distal humerus and right transhumeral amputations as the tumor was not responsive to the neoadjuvant chemotherapy. She was unable to complete adjuvant chemotherapy because her condition worsened with bacteremia and fungemia. Follow-up CECT thorax showed multiple lung lesions, the largest measuring around 3.3 cm at the right oblique fissure (Figure 4). She initially planned for PM, but intraoperatively, the decision was abandoned after finding new multiple lung nodules over the right middle and lower lobes in different sizes as well as multiple telangiectatic vessels (Figure 5). She was subsequently referred to palliative care for continuation of care.

Case 4

**Figure 6:**
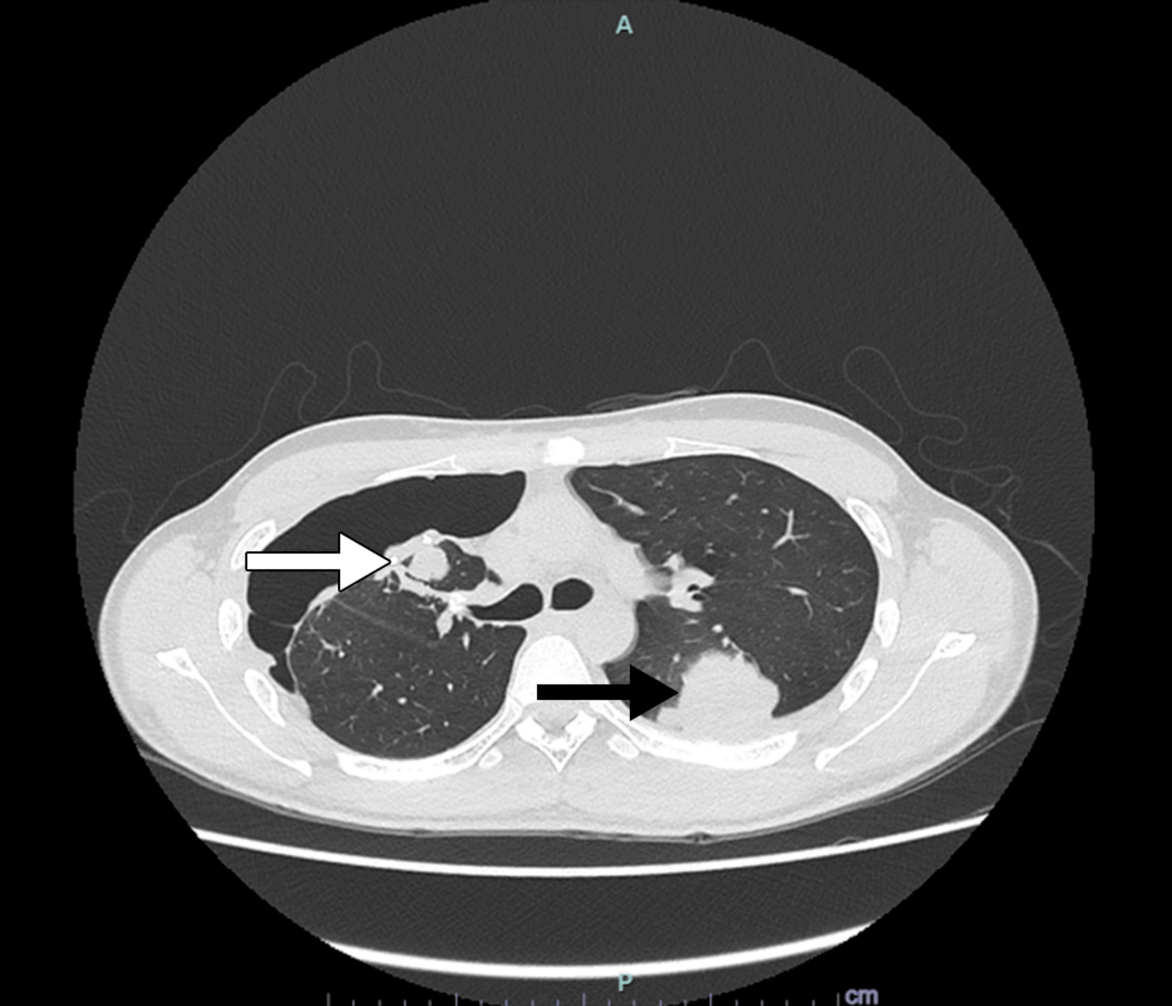
An axial view of the CT thorax showing lung nodules in the left lower lobe (black arrow) as well as the collapsed right upper lobe (white arrow) with right pneumothorax.

A 26-year-old gentleman has a history of left distal femur chondroblastic osteosarcoma. He underwent a left AKA and subsequently underwent two metastasectomies bilaterally separately, all three surgeries within the span of two years. A follow-up CT thorax revealed recurrent lung nodules, seen on the right upper lobe, right middle lobe, and left lower lobe, with pneumothorax over the right lung (Figure 6). After a thorough discussion, the patient wanted a third bilateral metastasectomy. A metastatectomy was done for the right lung, with both the nodules in the right upper and middle lobes resected, but the left lung was spared as the lung nodule was found to be too close in proximity to the left lower bronchus. The patient was referred to palliative care for continuation of care. He sadly passed away four months later.

## Discussion

The four cases presented above show that managing pulmonary metastases in sarcoma remains challenging. They can present with a wide range of progression and prognosis, requiring differing management strategies involving multidisciplinary teams.

After the landmark pulmonary metastasectomy case in 1939 by Barney and Churchill, pulmonary metastasectomy has gained recognition as a viable treatment option associated with improved long-term survival [6,7]. In the context of sarcoma with pulmonary metastases, despite the lack of randomized trials, existing evidence is in support of the surgical resection of pulmonary metastases secondary to sarcoma [8].

Pulmonary metastasectomy is the mainstay treatment for osteosarcoma with lung metastases. Multiple studies have demonstrated significant survival benefits, with complete surgical resection being the main prognostic factor affecting survival [8,9]. Observational studies have shown promising results with PM, but these findings are limited by bias, particularly patient selection bias, due to the retrospective nature of these studies. Neoadjuvant chemotherapy, adjuvant radiotherapy, and aggressive surgical resection with the aim of R0 resection and preservation of uninvolved pulmonary parenchyma further improve the prognosis for these patients [9].

Recurrence rates after PM are high, ranging from 30.6 to 69% [9]. Although studies have confirmed the correlation between an increasing number of lung nodules and decreased survival, there is no consensus on the threshold for considering a patient inoperable [5]. There is also no consensus on the size of the metastases before a patient is deemed inoperable. In general, the increased tumor burden negatively impacts resectability and survival. There is little evidence of the benefits of metastasectomy versus segmentectomy in such a disease. There is also a lack of data on the benefits of total thoracic lymphadenectomy in this context [8,9].

The past two decades have seen significant advancements in drug therapy for various cancers. The emergence of stereotactic body radiation therapy (SBRT) in the oncological field has revolutionized the landscape of local control for pulmonary metastases and has shown early favorable results in local ablation of pulmonary metastases [10-12]. It is crucial to carefully evaluate the available treatment options, considering the limitations associated with PM and the potential benefits of SBRT. This assessment should consider factors such as disease progression, patient preferences, potential adverse effects, etc.

Moving forward, a comprehensive review and discussion with a multidisciplinary team can provide valuable insights into the most appropriate treatment approach for patients with osteosarcoma and pulmonary metastases.

## Conclusions

Sarcoma with pulmonary metastases remains challenging to manage due to the wide range of progression and prognosis. Surgical metastasectomy provides improved survival for patients with sarcoma with pulmonary metastases. However, there is still no consensus on when a pulmonary metastasis is considered inoperable. With the emergence of new treatment methods, the management of sarcoma with pulmonary metastases is rapidly evolving. Thus, a case-by-case assessment and a multi-disciplinary team approach are paramount in deciding the best course of action for each patient.
